# Development and Evaluation of Health Recommender Systems: Systematic Scoping Review and Evidence Mapping

**DOI:** 10.2196/38184

**Published:** 2023-01-19

**Authors:** Yue Sun, Jia Zhou, Mengmeng Ji, Lusi Pei, Zhiwen Wang

**Affiliations:** 1 School of Nursing, Peking University Beijng China; 2 Wuhan Design and Engineering College Wuhan China

**Keywords:** health recommender systems, systematic review, evidence map, scoping review, recommender system

## Abstract

**Background:**

Health recommender systems (HRSs) are information retrieval systems that provide users with relevant items according to the users’ needs, which can motivate and engage users to change their behavior.

**Objective:**

This study aimed to identify the development and evaluation of HRSs and create an evidence map.

**Methods:**

A total of 6 databases were searched to identify HRSs reported in studies from inception up to June 30, 2022, followed by forward citation and grey literature searches. Titles, abstracts, and full texts were screened independently by 2 reviewers, with discrepancies resolved by a third reviewer, when necessary. Data extraction was performed by one reviewer and checked by a second reviewer. This review was conducted in accordance with the PRISMA-ScR (Preferred Reporting Items for Systematic Reviews and Meta-Analyses extension for Scoping Reviews) statement.

**Results:**

A total of 51 studies were included for data extraction. Recommender systems were used across different health domains, such as general health promotion, lifestyle, and generic health service. A total of 23 studies had reported the use of a combination of recommender techniques, classified as hybrid recommender systems, which are the most commonly used recommender techniques in HRSs. In the HRS design stage, only 10 of 51 (19.6%) recommender systems considered personal preferences of end users in the design or development of the system; a total of 29 studies reported the user interface of HRSs, and most HRSs worked on users’ mobile interfaces, usually a mobile app. Two categories of HRS evaluations were used, and evaluations of HRSs varied greatly; 62.7% (32/51) of the studies used the offline evaluations using computational methods (no user), and 33.3% (17/51) of the studies included end users in their HRS evaluation.

**Conclusions:**

Through this scoping review, nonmedical professionals and policy makers can visualize and better understand HRSs for future studies. The health care professionals and the end users should be encouraged to participate in the future design and development of HRSs to optimize their utility and successful implementation. Detailed evaluations of HRSs in a user-centered approach are needed in future studies.

## Introduction

Information and communication technologies provide new ways of searching and gathering health information. Health consumers have access to different kinds of resources that are disseminated through the World Wide Web [1]. The emergence and popularization of the internet have brought tremendous amounts of information to individuals, leading to serious information overload problems [1,2]. Meanwhile, people can obtain numerous health promotion intervention guidelines by searching for information, which helps them adopt a healthy lifestyle and independently manage their health behaviors. Although these interventions have been shown to be effective [3], they are not for everyone because people tend to exhibit a high degree of variability [4]. Hence, information overload and irrelevant information are major obstacles for drawing conclusions on personal health status and undertaking adequate actions. To solve these problems, it is important to develop new technologies that can be used to solve geographical access problems, deliver timely interventions, reduce intervention costs, and even help users exert better control over the intervention [5].

As technology evolves, some new ways to implement tailored interventions are being adopted. One such promising approach to computer-based tailored health interventions is the use of recommender systems. In the last decade, recommender systems have gained popularity, have been applied in several domains (eg, e-commerce, social media, and advertising) [6], and have proven useful in innumerable applications. Currently, these machine-based learning and information retrieval health recommender systems (HRSs) have the potential to predict items that will be relevant (eg, a health message) for individuals [7]. HRSs seem to have the potential to aid computer-tailored interventions by enhancing the user experience, as their recommendations for computer-tailored interventions are based on the user’s profile, and they select the best that can be recommended; they can be highly personalized and are most likely to be useful.

A scoping review, as a preliminary assessment of potential size and scope of available research on a topic, aims to identify the nature and extent of research evidence [8]. Evidence mapping is a useful methodology to overview available research about broad knowledge areas. We used evidence maps to represent the volume of work in different content areas; maps can provide an organized and understandable presentation of a large body of research [9]. Thus, to better understand the development and evaluation of HRSs as well as identify and map the state of the evidence, we conducted a systematic scoping review of existing research and created an evidence map.

This systematic review responds to the following research questions:

- What are the basic characteristics of the published HRSs？
- Which recommender techniques are being used in HRSs?
- What types of user interfaces are used in HRSs？
- Whether and how users were involved in the development of HRSs?
- How many types of HRS are evaluated, and are the end users involved in their evaluation?

## Methods

The conduct of this scoping review was based on the framework and principles reported by Arksey and O’Malley [10] and guided by the PRISMA-ScR (Preferred Reporting Items for Systematic reviews and Meta-Analyses extension for Scoping Reviews) guidelines [11]. A scoping review provides a literature overview by mapping key concepts in the evidence base of the research field, which can be used to inform needs and identify knowledge gaps [12]. The review included the following 5 key phases [10]:

- Stage 1: identifying the research questions
- Stage 2: identifying relevant studies
- Stage 3: study selection
- Stage 4: charting the data
- Stage 5: collating, summarizing, and reporting the results

### Data Sources

A comprehensive search strategy was developed by information and health specialists using a combination of Medical Subject Headings terms and free-text terms. The 6 databases of PubMed, Web of Science, Embase, Association for Computing Machinery, IEEE Xplore, and ScienceDirect were searched from the earliest record up to June 30, 2022. Electronic searches were conducted using the following keywords: (“recommender systems”) OR (“recommender system”) OR (“recommendation systems”) OR (“recommendation system”) AND (health OR patient OR patients). In addition, relevant studies were obtained by manual search of reference lists of all available records and conference proceedings in the initial search. The example of the search process can be found in Multimedia Appendix 1.

### Inclusion and Exclusion Criteria

Inclusion criteria for eligible studies were as follows: (1) studies that described or implemented HRSs whose primary focus was to improve health; (2) studies reporting on the targeted user; (3) studies published in English or Chinese; and (4) peer-reviewed publications. Studies with the following criteria were excluded from this review: (1) not detailed or not clearly reported the recommendations of HRS; (2) the full text was unavailable; (3) duplicate publications or secondary analysis of the same study; (4) technical reports and reviews; and (5) studies in other domains of knowledge.

### Study Selection Process

We imported the retrieved records into EndNote X9 (Clarivate) for management. Based on the preestablished inclusion and exclusion criteria, 2 reviewers initially screened the titles and abstracts. The full texts of the articles included were independently assessed. If the 2 reviewers did not reach consensus, a third reviewer decided whether the study should be included. We used consensus to resolve disagreements concerning selection and inclusion.

### Data Extraction

A data extraction form was developed to facilitate electronic comparison of entry, and we randomly selected 10 studies to test and refine it. The data extraction form included the following details: author name, year of publication, study origin (ie, country), target population, and HRS details (eg, recommended items, recommender techniques, user interface, and evaluation approach). Two reviewers reviewed all studies that met the inclusion criteria and extracted relevant data. Disagreements were resolved by discussion among the reviewers. The interrater agreement between 2 raters for full-text selection was evaluated and quantified with Cohen *κ*. Cohen *κ* was interpreted according to Altman’s definition, as follows: *κ*<2 as poor, 0.2<*κ*<0.4 as fair, 0.41<*κ*<0.60 as moderate, 0.61<*κ*<0.80 as good, and 0.81<*κ*<1.00 as excellent.

### Data Analysis (Mapping the Evidence)

Data extracted from primary studies were mapped to visually summarize outcome measures identified and coded by domain using the taxonomy proposed by Hors-Fraile et al [13]. We grouped studies by the type of HRSs being tested in Table 1. We summarized the results using a narrative descriptive synthesizing approach and presented them in tables and figures. For documenting the evidence characteristics, we used evidence maps to present outcome measures of HRSs.

**Table 1:** Taxonomy of health interventions using health recommender systems.

Characteristics		Description of all the extracted data	
**Basic information**			
	Therapeutic area		The target respective health domains
	Target population		Description of the user
	Publication year		Year when the study was conducted
	Country		Country or region where the study was conducted
**Health domains**			
	General health promotion		Yes or no
	Lifestyle		Yes or no
	Generic health service		Yes or no
	Other		Yes or no
**Recommender techniques**			
	Collaborative filtering^a^		Yes or no
	Content-based filtering^b^		Yes or no
	Knowledge-based filtering^c^		Yes or no
	Hybrid recommender system^d^		Yes or no
	Comparison between techniques		Yes or no
	Other		Yes or no
**User interface**			
	Mobile		Yes or no
	Web		Yes or no
	Other		Yes or no
**Evaluation approach**			
	Used metrics to assess performance (no user)		Metrics can be technical (eg, precision, accuracy, performance, recall, mean absolute error, NDCG^e^, simulation, root mean square error, *F*_1_-score, effectiveness, robustness, sensitivity, mean average, precision, and cosine similarity)
	Evaluations involving end user^f^		Safety; clinical effectiveness; patient perspectives; economic aspects; organizational aspects; sociocultural, ethical, and legal aspects

^a^Collaborative filtering recommendation system is designed to provide item recommendations to users based on users’ past behavior, by means of the ratings the user scored to the items he or she consumed.

^b^Content-based recommendation system obtains the user’s interest preference according to the user’s historical behavior and recommends items that are similar to other items preferred by the specific user.

^c^Knowledge-based filtering is another technique that incorporates knowledge by logic inference; it uses explicit knowledge about an item, user preferences, and other recommendation criteria.

^d^The hybrid recommendation systems were proposed to optimize the algorithms and address the limitations by combining 2 or more recommendation algorithms or introducing other algorithms.

^e^NDCG: normalized discounted cumulative gain.

^f^The domains are classified based on Model for Assessment of Telemedicine applications.

## Results

### Search Results of Included Studies

**Figure 1:**
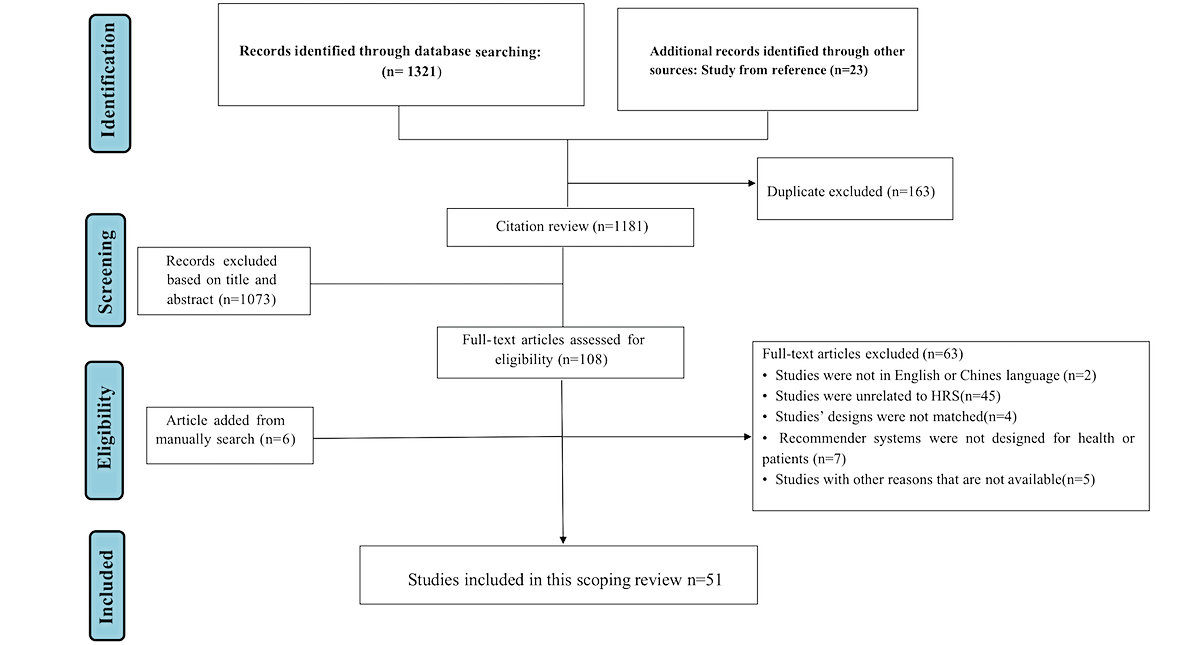
Flow diagram. HRS: health recommender system.

In this review, 1321 titles were retrieved, and after deduplication, 1181 abstracts were reviewed by 2 independent reviewers. A total of 108 articles were selected for full-text review, and 51 studies were included in this review (Multimedia Appendix 2 [14-65]). Interrater agreement between the 2 authors was excellent (*κ*=0.81) for the full-text selection. The detailed screening process is illustrated in Figure 1.

**Figure 2:**
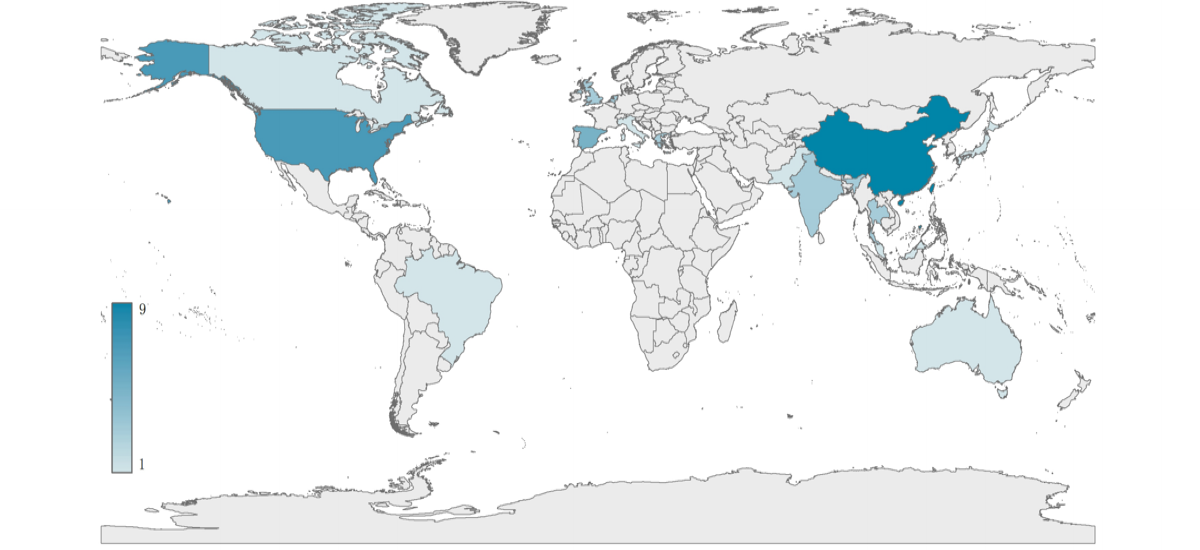
Distribution of the included articles in geographical map. A total of 51 studies were conducted in16 different countries, including China, United States, Spain, United Kingdom, Thailand, Italy, Japan, Korea, Malaysia, Netherlands, Pakistan, Singapore, Spain, Brazil, Australia, and Canada.

Regarding geographical distribution (Figure 2 and Multimedia Appendix 3), the included studies originated from 16 different countries, with major contributors being China (n=14, 27.5%), United States (n=10, 19.6%), Spain (n=4, 7.8%), and United Kingdom (n=3, 5.9%). Over the 13-year period that spanned from the oldest to the most recent study included in this review, there was a rising trend of relevant publications, with 1 discrete peak year or period—2017 (8 studies, 15.7%). Notably, more than half of the studies were published over the last 5 years.

### Recommended Items and User Interface

**Figure 3:**
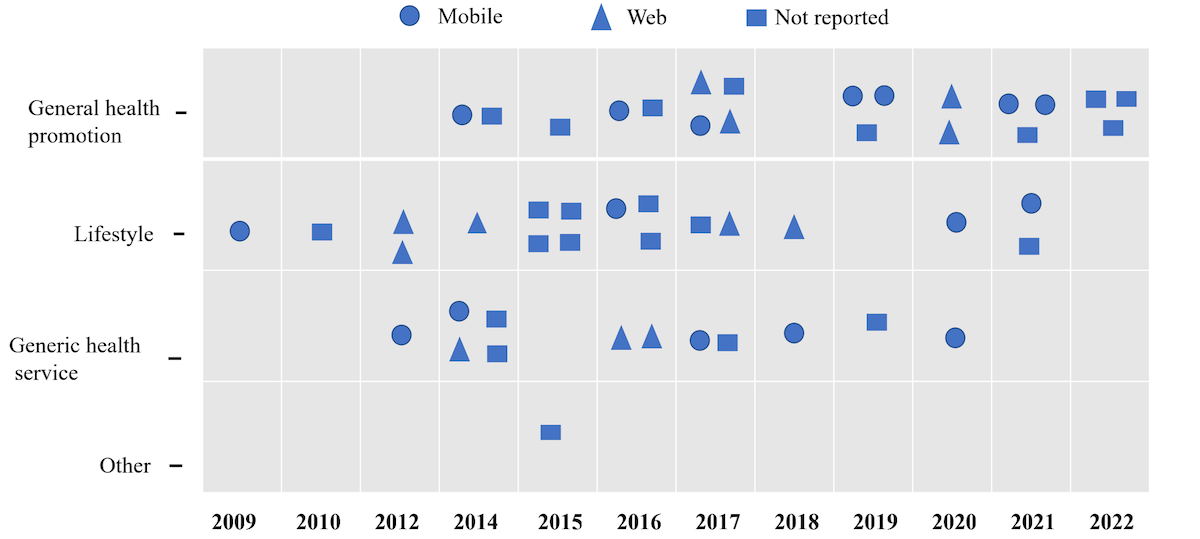
Effect of health recommender systems by year of publication and health domains.

In all 51 eligible studies, the health domains included general health promotion, lifestyle, generic health service, and some other domains. Most of the studies focused on lifestyle (n=17, 33.3%) or general health promotion (n=20, 39.2%). Other HRSs are used to treat generic health services (n=13, 25.5%; Figure 3). A large proportion of user interfaces were mobile, including mobile apps or web interfaces to show the recommended items (n=17, 33.3%); 12 (23.5%) studies were websites; and 22 (43.1%) studies did not report the interface used (Figure 3 and Multimedia Appendix 3). In 22 (43.1%) studies, the HRSs reported that they considered the user’s characteristics or preferences, and they recommended tailored messages.

### Recommender Techniques

**Figure 4:**
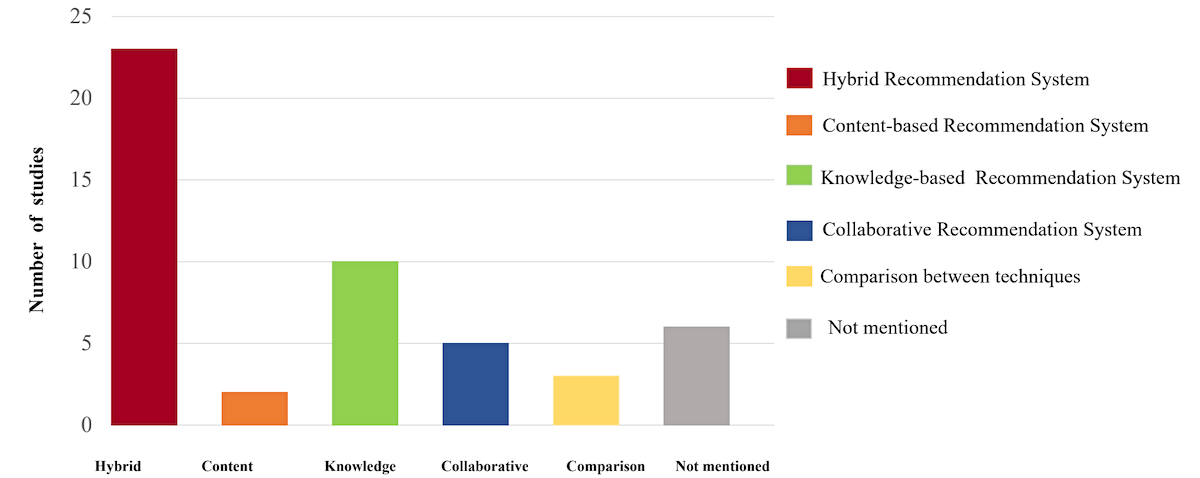
The number of recommender techniques used in health recommender systems.

The frequency of recommender techniques used in HRS types is presented in Figure 4. A total of 23 (45.1%) studies reported the use of hybrid recommendation systems, which are the most commonly used recommender type in HRSs. A group of 10 (19.6%) studies used knowledge-based filtering HRSs. Although collaborative filtering and content filtering are popular techniques, they were not used frequently in the HRS domain (n=5, 9.8%). Only 2 HRSs (n=2, 3.9%) relied on content-based filtering, and 3 studies (n=3, 5.9%) reported comparing different HRSs techniques to find the best algorithm.

### Users Involved in the Development of HRSs

Of all 51 eligible studies, 19.6% (10/51) recruited users in the development of HRSs. End users were recruited in the design phase of HRSs in 7 (13.7%) studies. In 5.9% (3/51) of the studies, the HRSs reported that end users participated in the test phase of HRSs (Multimedia Appendix 3).

### Evaluation Approach

In this study, we found 2 types of HRS evaluation approaches: (1) offline evaluations using computational methods and (2) evaluations involving an end user.

### Offline Evaluations Using Computational Methods

**Figure 5:**
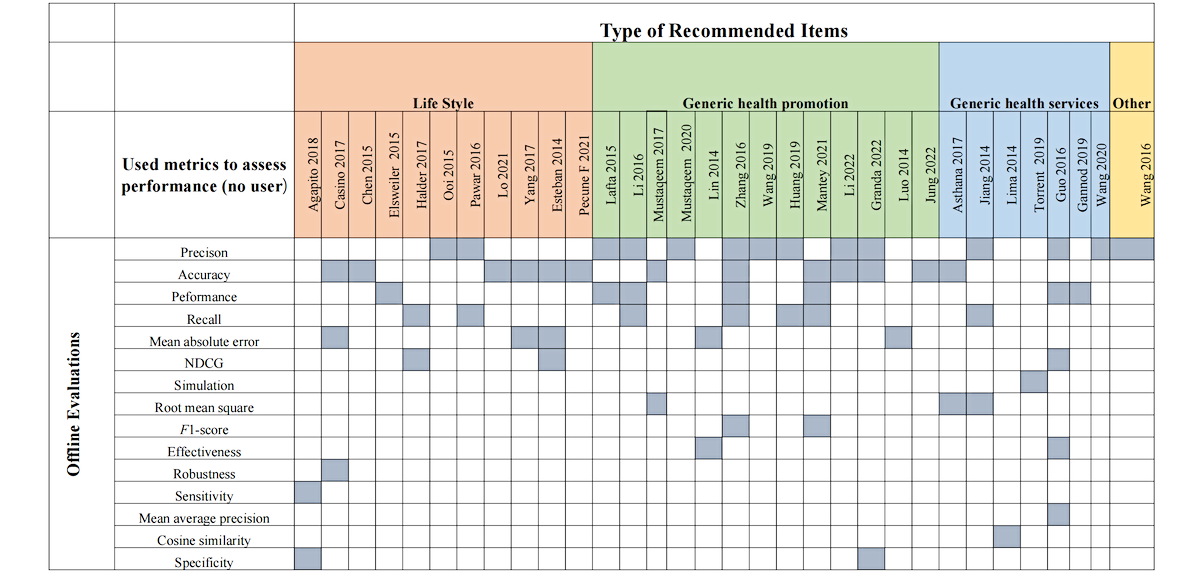
Overview of offline evaluations using computational methods in the studies. NDCG: normalized discounted cumulative gain [14,15,18,19,21,22,25,27-32,34,36,39,40,42,45,48-52,56-58,60-63,65].

In our study, 62.7% (32/51) of included studies used the metrics to assess performance. Precision (13/32, 40.7%), accuracy (12/32, 37.5%), performance (7/32, 21.9%), and recall (8/32, 25%) were the most used metrics among offline evaluation metrics. Other popular offline evaluation metrics are accuracy-related measurements, such as the mean absolute (percentage) error (5/32, 15.6%), root mean square error (3/32, 9.4%), normalized discounted cumulative gain (3/32, 9.3%), and *F*_1_-score (2/32, 6.2%). The details are illustrated in Figure 5.

### Evaluations Involving an End User

**Figure 6:**
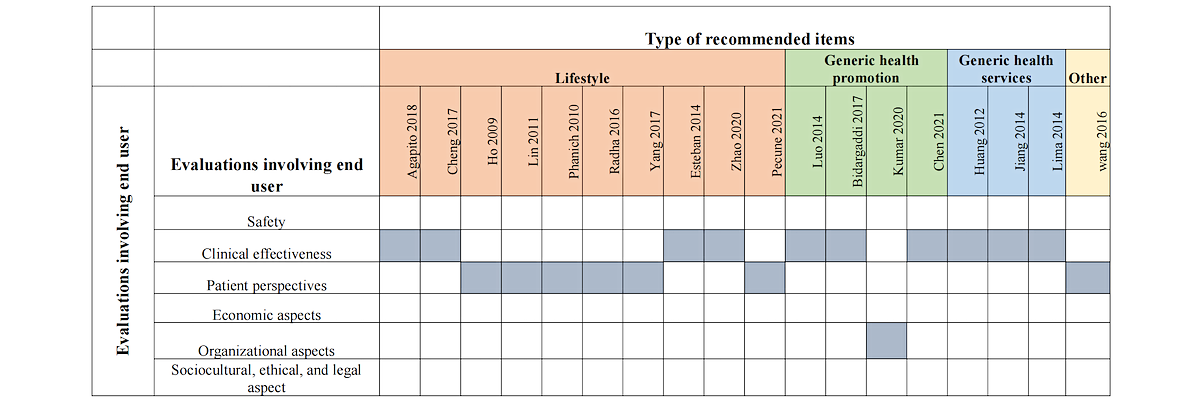
Overview of the user-involved evaluation used in the studies [14,20,23-25,29,31,35,37,38,40,42,47,48,53,54,59,61].

Of the total studies, 35.3% (18/51) included participants in their HRS evaluation. Clinical effectiveness (10/18, 55.6%) and patient perspectives (7/18, 38.9%) were the most commonly evaluated domains. Only 1 (2.2%) study used randomized controlled trial to evaluate organizational aspects. The details are illustrated in Figure 6.

## Discussion

### Principal Findings

Our study is the first to conduct a systematic scoping review and create an evidence map to give an overview of the state of the evidence on development and evaluation of HRSs. A total of 51 studies of HRSs were included in our systematic review, and the health domains included general health promotion, lifestyle, generic health service, and some other domains.

The main goals of HRSs are to retrieve trusted health information from the internet, to analyze what is suitable for the user profile and to select the best that can be recommended. Recent trends in health information seeking and developments in the fields of personal health records motivate our proposed approach for HRSs [13]. HRSs’ tailoring information to individual needs can better support patients in their search and retrieval efforts for securing appropriate information [66]. In our review, we found that only 10 studies recruited users and asked them to be involved in HRS development. For example, in the study conducted by Bravo-Torres [16], the design was developed following recommendations given by 25 end users. Moreover, to verify the operation and use of the application, several tests were carried out on a total of 25 older adults. The preferences and tastes of the participants were collected, and the information was stored in a database [67]. The authors aimed to influence their users’ health by improving the quality of the system, leading to better health outcomes, so the end user is always involved in both the design and evaluation processes. In our review, only 2 studies tested their HRSs by experts [25,41]. Experts such as clinicians, technicians, nurses, pharmacists, and therapists possess the right sets of knowledge and skills, and they interact with consumers regularly and are better aware of their concerns [68]. Thus, another way to improve the quality of HRSs is to consider expert reviews of HRSs and include their assessment data in the design and evaluation phases.

Our systematic review explored how many techniques were used for building recommendation systems. The most popular ones are hybrid recommender systems [69], collaborative filtering [70,71], and knowledge-based systems [72]. The so-called hybrid recommender systems are usually based on the main types of recommender systems. Most of the identified HRSs were hybrid types, mostly combining content-based filtering with collaborative filtering [73]. They can make more accurate predictions, as they are better for solving the issues where there is an abundance of data. For example, Agapito et al [14] proposed a web-based HRS (DIET Organizer System) that automatically provides users with health profiles including chronic kidney disease, diabetes, and hypertension status. In addition, based on the user’s health profile, this HRS provided individualized nutritional recommendations, with attention to food geographical origin [14]. Collaborative filtering is called the most mature and the most commonly implemented in other domains. Simply put, the basic task of collaborative filtering analyzes big groups of people and aims at finding much smaller sets of users who share their preferences with the user of interest. Maybe due to abundance of data in the health field, it is not commonly used in the health field, and only 5 studies showed relevance in the items recommended using collaborative ﬁltering. Li et al [60] used collaborative filtering to provide patients with drug recommendations. By creating a patient file, this file can be compared with other patient files with similar characteristics to achieve recommendations [60]. However, in 3 studies, the researchers compared different recommender techniques [42,74,75]. They aimed to find the best algorithm for a specific data set or end users.

With the increasing popularity of mobile devices and the development of wireless communication network technologies, an increasing number of studies integrate mobile devices and context-aware technology to develop HRSs. A total of 29 studies reported the user interface of HRSs; most HRSs worked on users’ mobile interfaces, usually a mobile app. Owing to the penetration, processing, connectivity capabilities, and accessibility of mobile devices [76,77], they have grown in the medical field [77]. It has been shown that mobile apps have potential effects on health behavior change [78] and chronic disease management. Through a wide variety of smart mobile devices (eg, iPhone, Android, Blackberry, and iPad), users can browse large quantities of health information anytime anywhere to assist self-health management. In addition, web technology is popular for these HRSs. Web technology has advantages in cross platform specificity, which leads to the use of multiple clients with different hardware and operating systems. EI-Gayar et al [46] found that mobile-based apps are preferred to computer-based applications in patients with diabetes, and as this system was designed as a mobile app, it managed to attract nutritionists’ attention.

On the basis of the papers included in our study, it seems that there are 2 distinct phases of development and hence evaluation at these distinct phases. In the first approach, the authors use metrics to assess performance (no user). We found that there are large disparities in accuracy, testing, analysis, and unified evaluation types used in previous studies. For example, Casino et al [18] reported that they measure robustness but do not outline what they measure as robustness. Consequently, it is difficult for new researchers to fully comprehend or take advantage of the benefits of the methods presented in other HRSs. Technology-related metrics (ie, *F*_1_-score, precision, and accuracy) may be sufficient to justify use in real-world settings. In this sense, it is necessary to continue reporting results on the evolution of HRSs research using computational methods. Some studies [79-81] have investigated the problem of a lack of evaluation in many articles. A greater focus on technical aspects, such as using the correct terminology and describing the system comprehensively, will benefit other researchers and policy makers willing to build on the previous successful experiences. In addition to accuracy, a variety of other metrics should be taken into account when evaluating recommender systems, and many HRSs need to be tested to demonstrate any proposed evaluation approach [71].

In the second approach, only 33.3% (n=17) of studies reported the evaluations involving an end user. Most HRSs aim to influence their users’ health. Thus, end users should be involved in the test processes and implementation evaluation of an HRS. For example, Bidargaddi et al [47] reported on findings from a randomized controlled trial that was designed to test the efficacy of a guided recommendation service for readily available mobile mental health apps for young people aged 16-25 years. Reporting the health promotion and behavior change will make it easier to understand the robustness and fidelity of the HRS study. Researchers have noticed this lack of user participation before, and it has been identified as the main challenge in this field [13]. Therefore, we recommend that researchers evaluate their HRSs with actual end users. The Model for Assessment of Telemedicine applications provides a structure for the multidisciplinary assessment of telemedicine applications by decision makers to choose the most efficient and cost-effective technologies with regard to the characteristics of the application, safety, clinical effectiveness, patient perspectives, as well as economic, organizational, sociocultural, ethical, and legal aspects [82]. Although there is immense potential in the use of HRS in health interventions, there is little information specifically on the effectiveness and organizational aspects of it thus far, indicating the need for further studies to address other domains. There are general frameworks that cover health information technology, such as the user-centric framework proposed by Knijnenburg et al [83]. However, future HRS studies should cover at least all aspects proposed in their taxonomy when disseminating their results. Moreover, the challenge is to add appropriate health or human behavior parameters into HRS frameworks or evaluation rules.

### Considerations for Future Research

First, although machine learning algorithms are difficult to explain for medical professionals, it is necessary to report or disclose this detailed information to validate the results and facilitate future research [5]. Second, end users and health care professionals should be encouraged to participate in the design and test phases of the HRSs. In addition, there is a need for further studies to evaluate these domains, including clinical effectiveness, patient perspectives, as well as economic, organizational, sociocultural, ethical, and legal aspects. Third, there is a lack of papers that include both clinical trials and simulations in the experimentation process, even though this would be beneficial. Researchers may focus on individual domains in articles depending on the research question and the word count limitations set by specific journals. It is recommended that the proposed model or framework for assessment of HRSs are applied as a complete framework, which will help researchers in choosing the most efficient and cost-effective technologies. Last, although the use of recommender systems has the potential to contribute to tailored health interventions, it is still sparse in the health domain. Researchers are encouraged to propose and support studies pertaining to HRSs in therapeutic areas other than generic health services and lifestyle.

### Strengths and Limitations

This is the first systematic scoping review and evidence map existing research in HRSs. As such, the study offers some important insights into the condition of recommender systems in the health field with a focus on technical aspects. Moreover, our findings can be useful in prioritizing areas of further implementation research in HRSs. This review also has some limitations. First, although we searched most health care information journals, we only included studies in English and Chinese languages, and some grey literature may have been excluded. The restriction to the search string and language may impact the included results. However, as this is a scoping review without synthesis of the evidence, we present the outcomes as reported by the authors of primary studies, and therefore, do not make determinations or recommendations as to the appropriateness or utility of outcomes. Second, some research may have implicitly reported computer-generated health advice in their studies. However, in these studies, they did not mention recommendation systems and were excluded from this review.

### Conclusions

HRSs have been increasingly used in recent years and may have significant potential to improve population health. There is a lack of scientific evidence in user-centered evaluation approaches, and some other metric parameters are ambiguous in HRS evaluation. The end users and professionals should be encouraged to participate in the design and development of HRSs to optimize their utility and successful implementation. This review can help nonmedical professionals and policy makers visualize and better understand HRSs in future studies.

## Appendix

Multimedia Appendix 1 Pubmed search strategy.

Multimedia Appendix 2 Studies included in the review.

Multimedia Appendix 3 Basic information of included studies.

## Abbreviations

HRS: health recommender system
PRISMA-ScR: Preferred Reporting Items for Systematic Reviews and Meta-Analyses extension for Scoping Reviews
