# Supplementary material for: Development and Evaluation of Health Recommender Systems: Systematic Scoping Review and Evidence Mapping
Source: J Med Internet Res. 2023 Jan 19;25:e38184. doi: 10.2196/38184 (PMC9896351; doi:10.2196/38184)
Supplement: Multimedia Appendix 1 [file jmir_v25i1e38184_app1.docx]

**Multimedia Appendix 1.**

**Pubmed search strategy**

#1 Search (recommender systems [Title/Abstract]) OR (recommender system [Title/Abstract]) OR (recommendation systems [Title/Abstract]) OR (recommendation system [Title/Abstract])

#2 Search (health [Title/Abstract]) OR (healthcare [Title/Abstract]) OR (patients [Title/Abstract]) OR (patient [Title/Abstract])

#3 #1 AND #2

**ACM search strategy**

When using to the ACM digital library, this query was adapted to the database library as follows: (“recommendation system” “recommender systems” “recommender system” “recommendation systems”) + (“patients” “patient” “health” “healthcare”).

Similar searches were performed in the remaining databases.
