# Supplementary material for: Development and Evaluation of Health Recommender Systems: Systematic Scoping Review and Evidence Mapping
Source: J Med Internet Res. 2023 Jan 19;25:e38184. doi: 10.2196/38184 (PMC9896351; doi:10.2196/38184)
Supplement: Multimedia Appendix 2 [file jmir_v25i1e38184_app2.docx]

**Multimedia Appendix 2 : Included reference**

14. Agapito, G., Simeoni, M., Calabrese, B., Caré, I., Lamprinoudi, T., Guzzi, P. H., Pujia, A., Fuiano, G., & Cannataro, M. (2018). DIETOS: A dietary recommender system for chronic diseases monitoring and management. Computer methods and programs in biomedicine, 153, 93–104. https://doi.org/10.1016/j.cmpb.2017.10.014

15. Asthana, S., Megahed, A., & Strong, R. (2017). A Recommendation System for Proactive Health Monitoring Using IoT and Wearable Technologies. IEEE International Conference on Ai & Mobile Services. IEEE.

16. Bravo-Torres, J. F., JO Ordoñez-Ordoñez, Gallegos-Segovia, P. L., Vintimilla-Tapia, P. E., M López-Nores, & Y Blanco-Fernández. (2017). A context-aware platform for comprehensive care of elderly people: Proposed architecture. Electrical, Electronics Engineering, Information & Communication Technologies. IEEE.

17. Buhl, M., Famulare, J., Glazier, C., Harris, J., & Gerber, M. S. (2016). Optimizing multi-channel health information delivery for behavioral change. Systems & Information Engineering Design Symposium. IEEE.

18. Casino, F., Patsakis, C., Batista, E., F Borràs, & A Martínez-Ballesté. (2017). Healthy routes in the smart city: a context-aware mobile recommender. IEEE Software, 34(6), 42-47.

19. Chen RC, Ting YH, Chen JK, Lo YW. The Nutrients of Chronic Diet Recommended Based on Domain Ontology and Decision Tree. In: Conference on Technologies and Applications of Artificial Intelligence. 2015 Presented at: TAAI'15; November 20-22, 2015; Tainan, Japan. [doi: 10.1109/taai.2015.7407127]

20. Cheng CY, Qian X, Tseng SH, Fu LC. Recommendation Dialogue System Through Pragmatic Argumentation. In: 26th IEEE International Symposium on Robot and Human Interactive Communication. 2017 Presented at: RO-MAN'17; August 29-30, 2017; Lisbon, Portugal. [doi: 10.1109/roman.2017.8172323]

21. Elsweiler, D., & Harvey, M. (2015). Towards Automatic Meal Plan Recommendations for Balanced Nutrition. RecSys '15: Ninth ACM Conference on Recommender Systems. ACM.

22. Halder, K., Kan, M. Y., & Sugiyama, K. (2017). Health Forum Thread Recommendation Using an Interest Aware Topic Model. ACM (pp.1589-1598). ACM.

23. Ho, T. C. T., & Chen, X. (2010). ExerTrek: A Portable Handheld Exercise Monitoring, Tracking and Recommendation System. e-Health Networking, Applications and Services, 2009. Healthcom 2009. 11th International Conference on.

24. Huang, Y. F., Peng, L., Qiao, P., & Lin, J. S. (2013). A doctor recommendation algorithm based on doctor performances and patient preferences. International Conference on Wavelet Active Media Technology & Information Processing. IEEE.

25. Jiang, H., & Wei, X. (2015). How to find your appropriate doctor: An integrated recommendation framework in big data context. 2014 IEEE Symposium on Computational Intelligence in Healthcare and e-health (CICARE). IEEE.

26. Krishna, S., Prasanna, V. R., Swagath, S., & Valliyammai, C. (2015). A trust enhanced Recommender System for medicare applications. Sixth International Conference on Advanced Computing. IEEE.

27. Lafta, R., Ji, Z., Tao, X., Yan, L., & Tseng, V. S. (2016). An Intelligent Recommender System Based on Short-Term Risk Prediction for Heart Disease Patients. IEEE/WIC/ACM International Conference on Web Intelligence & Intelligent Agent Technology. ACM.

28. Li, J., & Kong, J. (2016). Cell phone-based diabetes self-management and social networking system for American Indians. 2016 IEEE 18th International Conference on e-Health Networking, Applications and Services (Healthcom). IEEE.

29. Lima-Medina, E., Loques, O., & Mesquita, C. (2014). "Minha Saude" a healthcare social network for patients with cardiovascular diseases. 2014 IEEE 3nd International Conference on Serious Games and Applications for Health (SeGAH). IEEE.

30. Lo, Y. W., Zhao, Q., Ting, Y. H., & Chen, R. C. (2015). Automatic generation and recommendation of recipes based on outlier analysis. IEEE International Conference on Awareness Science & Technology. IEEE.

31. Yan, L., Ling, C., Schuurman, J., & Petrella, R. (2014). GlucoGuide: An Intelligent Type-2 Diabetes Solution Using Data Mining and Mobile Computing. IEEE International Conference on Data Mining Workshop. IEEE.

32. Mustaqeem, A., Anwar, S. M., Khan, A. R., & Majid, M. (2017). A statistical analysis based recommender model for heart disease patients. International Journal of Medical Informatics, 108(dec.), 134-145.

33. Ntalaperas, D., Bothos, E., Perakis, K., Magoutas, B., & Mentzas, G. N. (2015). DISYS: an intelligent system for personalized nutritional recommendations in restaurants. PCI '15: 19th Panhellenic Conference on Informatics. ACM.

34. Ooi, C., Iiba, C., & Takano, C. (2015). Ingredient substitute recommendation for allergy-safe cooking based on food context. Communications, Computers & Signal Processing. IEEE.

35. Lin, Y., Jessurun, J., Vries, B. D., & Timmermans, H. (2011). Motivate: towards Context-Aware Recommendation Mobile System for Healthy Living. 5th International Conference on Pervasive Computing Technologies for Healthcare, PervasiveHealth 2011, Dublin, Ireland, May 23-26, 2011. IEEE.

36. Pawar, K. R., Ghorpade, T., & Shedge, R. (2016). Constraint based recipe recommendation using forward checking algorithm. 2016 International Conference on Advances in Computing, Communications and Informatics (ICACCI). IEEE.

37. Phanich, M., Pholkul, P., & Phimoltares, S. (2010). Food Recommendation System Using Clustering Analysis for Diabetic Patients. International Conference on Information Science & Applications. IEEE.

38. Radha, M., Willemsen, M. C., Boerhof, M., & Ijsselsteijn, W. A. (2016). Lifestyle recommendations for hypertension through Rasch-based feasibility modeling. ACM.

39. Torrent-Fontbona, Ferran, Lopez, & Beatriz. (2019). Personalized adaptive cbr bolus recommender system for type 1 diabetes. IEEE Journal of Biomedical and Health Informatics.

40. Wang SL, Chen YL, Kuo AM, Chen HM,Shiu SM, (2016). Design and evaluation of a cloud-based mobile health information recommendation system on wireless sensor networks. Computers & Electrical Engineering, 49(C), 221-235.

41. Wongpun, S., & Guha, S. (2017). Elderly care recommendation system for informal caregivers using case-based reasoning. 2017 IEEE 2nd Advanced Information Technology, Electronic and Automation Control Conference (IAEAC). IEEE.

42. Yang, L., Hsieh, C. K., Yang, H., Pollak, J. P., Dell, N., Belongie, S., Cole, C., & Estrin, D. (2017). Yum-Me: A Personalized Nutrient-Based Meal Recommender System. ACM transactions on information systems, 36(1), 7. <https://doi-org-443.webvpn.bjmu.edu.cn/10.1145/3072614>

43. Zaini, N., Latip, M., Omar, H., Mazalan, L., & Norhazman, H. (2012). Online personalized audio therapy recommender based on community ratings. IEEE Symposium on Computer Applications & Industrial Electronics. IEEE.

44. Zaman, N., & Li, J. (2014). Semantics-Enhanced Recommendation System for Social Healthcare. IEEE, 765-770.

45. Lin, Y. F., Hsin-Han, S., Yang, Y. C., & Vincent, T. (2014). Design of a real-time and continua-based framework for care guideline recommendations. International Journal of Environmental Research and Public Health, 11(4).

46. El-Gayar O, Timsina P, Nawar N, Eid W. Mobile applications for diabetes self-management: status and potential. J Diabetes Sci Technol 2013 Jan 01;7(1):247-262 [FREE Full text] [doi: 10.1177/193229681300700130] [Medline: 23439183]

47. Bidargaddi, N., Musiat, P., Winsall, M., Vogl, G., & Schrader, G. (2017). Efficacy of a web-based guided recommendation service for a curated list of readily available mental health and well-being mobile apps for young people: randomized controlled trial. Journal of Medical Internet Research, 19(5), e141.

48. Esteban, B., Á Tejeda-Lorente, Porcel, C., Arroyo, M., & Herrera-Viedma, E. (2014). Tplufib-web: a fuzzy linguistic web system to help in the treatment of low back pain problems. Knowledge-Based Systems, 67, 429-438.

49. Li, Gu, Bo, Ji, Cuili, & Ya, et al. (2016). Which doctor to trust: a recommender system for identifying the right doctors. Journal of Medical Internet Research, 18(7), e186.

50. Huang YJ. Design and Implementation of Personalized Health Information Service System [D]. Beijing University of Posts and Telecommunications, 2019.

51. Wang K. Research and implementation of personalized health knowledge recommendation system [D]. Hubei University of Technology, 2019. DOI: 10.27131/d.cnki.ghugc.2019.000005.

52. Mustaqeem, A., Anwar, S. M., & Majid, M. (2020). A modular cluster based collaborative recommender system for cardiac patients. Artificial intelligence in medicine, 102, 101761. https://doi.org/10.1016/j.artmed.2019.101761

53. Kumar, A., Aikens, R. C., Hom, J., Shieh, L., Chiang, J., Morales, D., Saini, D., Musen, M., Baiocchi, M., Altman, R., Goldstein, M. K., Asch, S., & Chen, J. H. (2020). OrderRex clinical user testing: a randomized trial of recommender system decision support on simulated cases. Journal of the American Medical Informatics Association : JAMIA, 27(12), 1850–1859. https://doi.org/10.1093/jamia/ocaa190

54. Zhao, Z., Arya, A., Orji, R., & Chan, G. (2020). Effects of a Personalized Fitness Recommender System Using Gamification and Continuous Player Modeling: System Design and Long-Term Validation Study. JMIR serious games, 8(4), e19968. https://doi.org/10.2196/19968

55. Jon, Hael, Brenas, Eun, Kyong, & Shin, et al. (2019). A hybrid recommender system to guide assessment and surveillance of adverse childhood experiences. Studies in health technology and informatics, 262, 332-335.

56. Wang, Z., Huang, H., Cui, L., Chen, J., An, J., & Duan, H., et al. (2020). Using natural language processing techniques to provide personalized educational materials for chronic disease patients in china: development and assessment of a knowledge-based health recommender system. JMIR Medical Informatics, 8(4).

57. Gannod, G. C., Kma, M., Haitsma, K. V., Martindale, B. N., & Heppner, B. A. (2018). A machine learning recommender system to tailor preference assessments to enhance person-centered care among nursing home residents. The Gerontologist.

58. Mantey EA, Zhou C, Anajemba JH, Okpalaoguchi IM, Chiadika OD. Blockchain-Secured Recommender System for Special Need Patients Using Deep Learning. Front Public Health. 2021 Sep 20;9:737269. doi: 10.3389/fpubh.2021.737269.

59. Chen, J., Houston, T. K., Faro, J. M., Nagawa, C. S., Orvek, E. A., Blok, A. C., Allison, J. J., Person, S. D., Smith, B. M., & Sadasivam, R. S. (2021). Evaluating the use of a recommender system for selecting optimal messages for smoking cessation: patterns and effects of user-system engagement. BMC public health, 21(1), 1749. https://doi.org/10.1186/s12889-021-11803-8

60. Li, X., Ma, D., Ren, Y., Luo, J., & Li, Y. (2022). Large-scale Prediction of Drug-Protein Interactions Based on Network Information. Current computer-aided drug design, 18(1), 64–72. https://doi-org-443.webvpn.bjmu.edu.cn/10.2174/1573409917666210315094213

61. Pecune, F., Callebert, L., & Marsella, S. (2022). Designing Persuasive Food Conversational Recommender Systems With Nudging and Socially-Aware Conversational Strategies. Frontiers in robotics and AI, 8, 733835. https://doi-org-443.webvpn.bjmu.edu.cn/10.3389/frobt.2021.733835

62. Granda Morales, L. F., Valdiviezo-Diaz, P., Reátegui, R., & Barba-Guaman, L. (2022). Drug Recommendation System for Diabetes Using a Collaborative Filtering and Clustering Approach: Development and Performance Evaluation. Journal of medical Internet research, 24(7), e37233. https://doi-org-443.webvpn.bjmu.edu.cn/10.2196/3723

63. Jung, Y. L., Yoo, H. S., & Hwang, J. (2022). Artificial intelligence-based decision support model for new drug development planning. Expert systems with applications, 198, 116825. <https://doi-org-443.webvpn.bjmu.edu.cn/10.1016/j.eswa.2022.116825>

64. Shao, M., Jiang, L., Meng, Z., & Xu, J. (2022). Computational Drug Repurposing Based on a Recommendation System and Drug-Drug Functional Pathway Similarity. Molecules (Basel, Switzerland), 27(4), 1404. https://doi-org-443.webvpn.bjmu.edu.cn/10.3390/molecules27041404

65. Zhang, W., Zou, H., Luo, L., Liu, Q., Wu, W., & Xiao, W. (2016). Predicting potential side effects of drugs by recommender methods and ensemble learning. Neurocomputing, 173(JAN.15PT.3), 979-987.
